# Clinical characterization of *int22h1/int22h2*-mediated Xq28 duplication/deletion: new cases and literature review

**DOI:** 10.1186/s12881-015-0157-2

**Published:** 2015-03-14

**Authors:** Ayman W El-Hattab, Christian P Schaaf, Ping Fang, Elizabeth Roeder, Virginia E Kimonis, Joseph A Church, Ankita Patel, Sau Wai Cheung

**Affiliations:** Department of Molecular and Human Genetics, Baylor College of Medicine, One Baylor Plaza, MS NAB 2015, Houston, TX 77030 U.S.A; Division of Clinical Genetics and Metabolic Disorders, Department of Pediatrics, Tawam Hospital, Al-Ain, United Arab Emirates; Jan and Dan Duncan Neurological Research Institute, Texas Children’s Hospital, Houston, TX USA; Section of Genetics, Department of Pediatrics, Baylor College of Medicine, Children’s Hospital of San Antonio, San Antonio, TX USA; Division of Genetics and Genomics, Department of Pediatrics, University of California, Irvine Medical Center, Orange, CA USA; Division of Clinical Immunology and Allergy, Children’s Hospital Los Angeles, and Keck School of Medicine, University of Southern California, Los Angeles, CA USA

**Keywords:** Chromosomal rearrangements, X-linked intellectual disability, Chromosomal microarray analysis

## Abstract

**Background:**

*Int22h1/int22h2*-mediated Xq28 duplication syndrome is caused by ~0.5 Mb chromosomal duplications mediated by nonallelic homologous recombination between intron 22 homologous region 1 (*int22h1*) and 2 (*int22h2*), which, in addition to *int22h3*, are also responsible for inversions disrupting the *F8* gene in hemophilia A. This syndrome has recently been described in 9 males with cognitive impairment, behavioral problems, and distinctive facial features; and 6 females with milder phenotypes. The reciprocal deletion was previously reported in a mother and daughter. It was suggested that this deletion may not have phenotypic effects in females because of skewed chromosome X inactivation, but may be embryonic lethal in males.

**Methods:**

Array comparative genomic hybridization analyses were performed using oligonucleotide-based chromosomal microarray. Chromosome X inactivation studies were performed at the *AR* (androgen receptor) and *FMR1* (fragile X mental retardation 1) loci.

**Results:**

We present here 5 males and 6 females with *int22h1/int22h2*-mediated Xq28 duplication syndrome. The males manifested cognitive impairment, behavioral problems, and distinctive facial features. Two of the six females manifested mild cognitive impairment. This duplication was maternally inherited, and skewed chromosome X inactivation was observed in the majority of females carrying the duplication. We also report the reciprocal deletion in a mother and daughter with overweight, but normal cognition. In addition, we present the first case of a prenatally diagnosed *de novo int22h1/int22h2*-mediated deletion in a healthy female infant. We reviewed individuals previously reported with similar or overlapping rearrangements and evaluated the potential roles of genes in the rearrangement region.

**Conclusions:**

The similarity of clinical features among individuals with the *int22h1/int22h2*-mediated Xq28 duplication supports the notion that this duplication causes a recognizable syndrome that affects males with females exhibiting milder phenotypes. It is suggested that the observed cognitive impairment in this syndrome results from increased dosage of *RAB39B* gene located within the duplicated region. Increased dosage of *CLIC2* may also contribute to the phenotype. The reciprocal deletion results in skewed chromosome X inactivation and no clinical phenotype in females. Review of overlapping deletions suggests that hemizygous loss of *VBP1* may be the cause for the proposed male lethality associated with this deletion.

## Background

Intellectual disability (ID), which affects 2% of the population, is diagnosed in individuals with significantly impaired intellectual function (Intelligence Quotient (IQ) of 70 or below), poor adaptive skills, and onset before 18 years of age [1]. X-linked ID (XLID) is estimated to account for 10% of ID in males and thus has a prevalence of 1:1000 males [2]. Although XLID is most often caused by single-gene defects, the use of array comparative genomic hybridization (array CGH) has led to the identification of chromosome X rearrangements in a significant number of individuals with XLID [3]. Not only deletions, but also duplications in chromosome X can result in XLID, supporting the concept that increased gene dosage can disrupt normal cognitive development. The most common XLID-related chromosomal rearrangements are duplications of Xq28 comprising the *MECP2* gene (153.3 Mb, hg19), which present with intellectual disability, distinctive facial features, hypotonia, seizures, and recurrent infections [4,5]. The detrimental effect of *MECP2* duplication was predicted by a mouse model in which *Mecp2* overexpression resulted in a progressive neurological disorder resembling the human disease [6].

We previously described a novel ~0.5 Mb duplication in Xq28 (154.1 - 154.6 Mb, hg19) located telomeric to the *MECP2* locus and mediated by nonallelic homologous recombination between low-copy repeats (LCRs) intron 22 homologous region 1 (*int22h1*) and 2 (*int22h2*), which, in addition to *int22h3*, are also responsible for inversions disrupting the *F8* gene in hemophilia A. This duplication was identified in four males with cognitive impairment who shared similar facial features, behavioral abnormalities, and recurrent infections suggesting that this duplication results in a novel recognizable XLID syndrome [7]. Subsequently, an identical duplication was described in a boy with developmental delay, Pierre-Robin sequence, and distinctive facial features [8]. Recently, four additional males with the same duplication were reported with cognitive impairment, behavioral problems, and distinctive facial features [9]. The frequency of *int22h1/int22h2*-mediated Xq28 duplication has been estimated at 1:1000 among males with ID [9]. These duplications were found to be inherited from mothers with skewed chromosome X inactivation (XCI) in the majority of the cases [7-9]. Clinical similarities among the individuals described in these three reports support that *int22h1/int22h2*-mediated Xq28 duplication results is a recognizable XLID syndrome.

The reciprocal *int22h-1/int22h-2*-mediated Xq28 deletion was also first reported by us in a girl and her mother both of whom exhibited normal cognition and skewed XCI. In addition, the mother had two spontaneous miscarriages during first trimester. It was suggested that this deletion has no phenotypic effect in females; however, it may be embryonic lethal in males resulting in higher miscarriage rates in females carrying this deletion [7].

In this report we describe 11 additional individuals (5 males and 6 females) from five different families with *int22h1/int22h2*-mediated Xq28 duplication. In addition, we report two families with the reciprocal deletion. We present a review of individuals reported with similar or overlapping rearrangements and discuss the potential roles of genes in the rearrangement region.

## Methods

### Array CGH

Array CGH analysis was performed at the Medical Genetics Laboratories (MGL) at Baylor College of Medicine (BCM). The study received the ethics approval from the Institutional Review Board (IRB) of BCM. Written informed consent approved by the IRB of BCM was obtained from the participants or their parents or legal guardians in the case of children to participate in the study and to publish the clinical details and any accompanying images. A copy of the written consents is available for review by the Editor of this journal. Array CGH tests were performed using oligonucleotide-based chromosomal microarray (CMA OLIGO) either version 7 or 8. CMA OLIGO version 7 comprises approximately 105,000 oligonucleotides, which cover the entire genome at an average resolution of 30 kb with increased coverage at known disease loci. The array also includes six regions of known polymorphic variants [10,11]. CMA OLIGO version 8 compromises approximately 180,000 oligonucleotides, which cover the entire genome at an average resolution of 30 kb with increased coverage at known disease loci. Exonic coverage for 1,700 selected disease-causing genes and 670 probes for mitochondrial genome are also included [10,12].

### Fluorescence *in situ* hybridization analyses

Confirmatory fluorescence *in situ* hybridization (FISH) analyses with bacterial artificial chromosome (BAC) clones were performed on peripheral blood lymphocytes using standard procedures following the detection of copy-number changes via array CGH [13].

### Chromosome X-inactivation studies

Chromosome X-inactivation (XCI) studies were performed at the *AR* (androgen receptor) and *FMR1* (fragile X mental retardation 1) loci [7,14,15]. The XCI ratio was calculated and inactivation ratios greater than 80:20 were designated as skewed XCI, whereas ratios greater than 95:5 were considered as extremely skewed XCI [16].

## Results

### Clinical description

Herein, we describe the clinical features in the members of families 1–5 with *int22h-1/int22h-2*-mediated Xq28 duplication and the members of families 6 and 7 with *int22h-1/int22h-2*-mediated Xq28 deletion.

**Figure 1:**
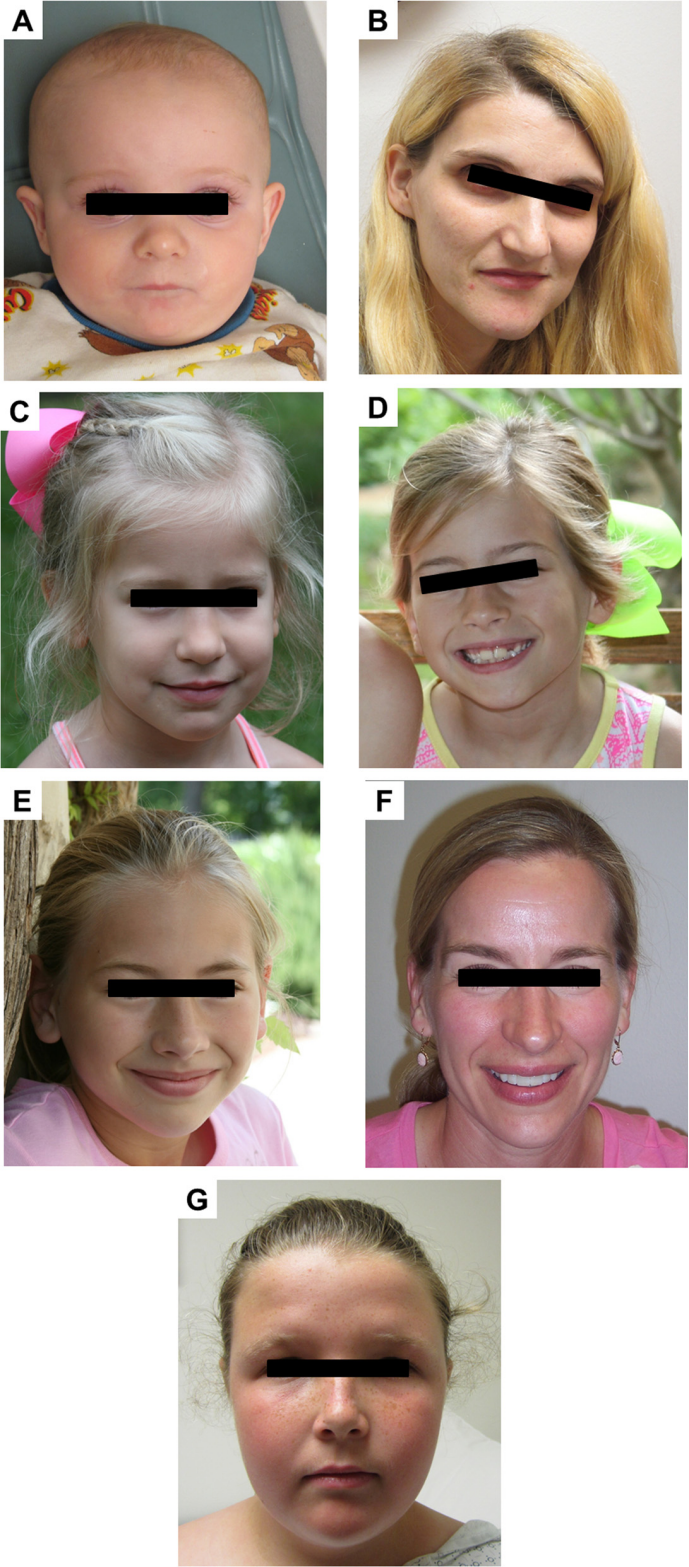
**Facial features in individuals with** ***int22h-1/int22h-2*** **-mediated Xq28 rearrangements. A**: facial features of the proband in family 1, including high forehead, sparse eyebrows and scalp hair, long eyelashes, upper eyelid fullness, broad and depressed nasal bridge, anteverted nares, and long philtrum. **B**: facial features of the mother in family 1, including deep set eyes and thick lower lip. **C**: facial features of the youngest sister in family 5 including high forehead, full upper eyelid, broad nasal bridge, and thick lower lip. **D**: facial features of the middle sister in family 5 including high forehead, full upper eyelid, broad nasal bridge, and thick lower lip. **E**: facial features of the oldest sister in family 5 including high forehead, full upper eyelid, and thick lower lip. **F**: facial features of the mother in family 5 including high forehead, elongated face, full upper eyelid, and thick lower lip. **G**: facial features of the proband in family 6, including high forehead, deep-set eyes, epicanthus, and broad nasal bridge.

Family 1 includes a 9-month-old male infant and his 25-year-old mother. The boy was born at term with an uncomplicated perinatal course and a birth weight of 3.3 kg. Shortly after birth, he was diagnosed with esophageal atresia with tracheoesophageal fistula for which he underwent surgical repair on the third day of life. During early infancy he was noticed to be developmentally delayed. At the age of 9 months he was unable to sit unsupported, pull to stand, or crawl; but he babbled, had a social smile, and was able to roll from front to back. His medical history was significant for recurrent episodes of upper respiratory tract infections. His physical examination demonstrated normal growth parameters, phimosis, and distinctive facial features (Figure 1A). His mother reported a personal history of learning disability with a need for special education. Her medical history was significant for cardiac valvular disease and scoliosis. She also had some distinctive facial features (Figure 1B). The boy was the only child for his parents, but had 5 maternal half siblings including a 5-year-old half-sister with developmental delay and an 8-year-old half-brother with developmental delay and attention deficit hyperactivity disorder (ADHD) (Table 1).

**Table 1:** **Clinical features of individuals with** ***int22h1/int22h2*** **-mediated Xq28 duplication**

		**El-Hattab** ***et al.*** **2011 (Ref** **[** 7 **]** **)**		**Lannoy** ***et al.*** **2013 (Ref** **[** 8 **]** **)**		**Vanmarsenille** ***et al.*** **2014 (Ref** **[** 9 **]** **)**		**Family 1**		**Family 2**			**Family 3**	**Family 4**	**Family 5**				**Total N = 26**	
		**4 M**	**3 M**	**1 M**	**1 F**	**4 M**	**2 F**	**Proband M**	**Mother**	**Older brother**	**Younger brother**	**Mother**	**Proband M**	**Proband M**	**Youngest sister**	**Middle sister**	**Oldest sister**	**Mother**	**14 M**	**12** **F**
Cognitive impairment		4/4	3/3	1/1	-	4/4	1/2	+	+	+	+	-	+	+	-	+	-	-	14/14	6/12
**Facial features**	High forehead	4/4	1/3	1/1	-	2/4	-	+	-	+	-	-	-	-	+	+	+	+	9/14	5/12
	Long face	1/4	-	1/1	-	1/4	-	-	-	-	-	-	-	-	-	-	-	+	3/14	1/12
	Sparse eyebrows	1/4	1/3	-	-	-	-	+	-	-	-	-	-	-	-	-	-	-	2/14	1/12
	Long eyelashes	-	-	-	-	-	-	+	-	-	-	-	-	+	-	-	-	-	2/14	-
	Upper eyelid fullness	4/4	-	1/1	-	-	-	+	-	-	-	-	+	+	+	+	+	+	8/14	4/12
	Deep seated eyes	2/4	-	-	-	1/4	-	-	+	-	-	-	-	-	-	-	-	-	3/14	1/12
	Broad nasal bridge	3/4	1/3	1/1	-	-	-	+	-	+	+	-	-	-	+	+	-	-	7/14	3/12
	Anteverted nares	-	-	1/1	-	1/4	-	+	-	-	-	-	-	-	-	-	-	-	3/14	-
	Thin upper lip	-	-	1/1	-	1/4	-	-	-	-	-	-	-	-	-	-	-	-	2/14	-
	Thick lower lip	3/4	1/3	-	-	-	-	-	+	-	+	-	+	-	+	+	+	+	5/14	6/12
	Micro/retrognathia	2/4	-	1/1	-	-	-	-	-	-	-	-	-	-	-	-	-	-	3/14	-
	Long philtrum	-	-	1/1	-	1/4	-	+	-	-	-	-	-	-	-	-	-	-	3/14	-
	Large ears	-	-	1/1	-	2/4	-	-	-	-	-	-	-	-	-	-	-	-	3/14	-
	Simple helices	1/4	-	-	-	1/4	-	-	-	-	-	-	-	-	-	-	-	-	2/14	-
**Tall stature**		1/4	-	-	-	1/4	-	-	-	-	-	-	+	-	-	-	-	-	3/14	-
**Obesity**		2/4	1/3	-	-	2/4	-	-	-	-	-	-	-	-	-	-	-	-	4/14	1/12
**Behavioral problems**		3/4	-	-	-	3/4	-	-	-	+	+	-	-	+	-	+	-	-	9/14	1/12
**Recurrent infections**		4/4	-	-	-	1/4	-	+	-	+	+	-	+	+	-	-	-	-	10/14	-
Atopic diseases*		2/4	-	-	-	1/4	-	-	-	+	-	-	+	+	-	-	-	-	6/14	-
**Congenital malformations**				Pierre Robin				EA/TEF	Cardiac valvular disease		ASD				hemihyper-plasia					

M: male, F: female, EA/TEF: esophageal atresia with tracheoesophageal fistula, ASD: atrial septal defect.

*Atopic diseases include asthma, allergic rhinitis, and eczema.

Family 2 includes two brothers and their mother. The older brother was 9 years old with developmental delay and behavioral problems. He was born at term with birth weight of 3.6 kg. Prenatal sonogram showed polyhydramnios, otherwise the pregnancy was uncomplicated. During the first year of life he was noticed to be developmentally delayed. He required special education classes and also had ADHD, aggressive behaviors (tantrums, hitting others and self), disruptive behavior at school, and autistic features. His medical history was significant for recurrent otitis media, eczema, and allergic rhinitis. His physical examination showed normal growth parameters, clinodactyly, and distinctive facial features including high forehead and broad nasal bridge. The younger brother was 6 years old with developmental delay. He was born at term with birth weight of 3.7 kg. As his brother, he was found to be developmentally delayed during infancy. He started walking at 18 months and had his single words at 2 years. At the of 6 years he was able to make 3–5 word sentences but was not toilet trained. He required special education and also had ADHD and aggressive behavior including hitting others and throwing objects. His medical history was significant for a small atrial septal defect, recurrent otitis media with tympanostomy tube placement, myopia, and astigmatism. His physical examination revealed normal growth parameters and distinctive facial features including full nasal tip, broad nasal bridge, and full lower lip. They had no other siblings. The mother was 35 years old. She graduated high school and completed 2 years of college with no history of learning difficulties or behavioral problems (Table 1).

Family 3 includes a 15-year-old boy who was born at term with uncomplicated perinatal course. During early childhood, he gained his developmental milestones appropriately. However, he was noticed to have learning difficulties starting in kindergarten. Formal evaluations showed dyslexia, dysgraphia, and impaired processing speed. He had difficulties in writing and reading and required special education. His medical history was significant for asthma, allergic rhinitis, and recurrent otitis media with tympanostomy tubes placement and adenotonsillectomy. His physical examination showed weight and height just above the 99^th^ percentiles and head circumference at 98^th^ percentile. He also had clinodactyly, mild kyphosis, and distinctive facial features including upper eyelid fullness, tubular nose, and thick lower lip (Table 1). He is the only child for his parents. His mother is 50 years old and reported a personal history of learning difficulties and dyslexia.

Family 4 includes a 12-year-old boy who was born at term. Both his parents used illicit substances. Therefore, at the age of 20 months, he was removed from his family and adopted. Since early childhood, he was noticed to be delayed. His IQ was found to be 59 at the age of 11 years and language evaluation revealed significant deficits in expressive language. He required special education and was diagnosed to have ADHD and bipolar disorder. In addition, he had impulsivity, irritability, and insomnia. His medical history was significant for recurrent otitis media and lower respiratory tract infections during the first 6 years of life. He also had asthma and allergic rhinitis. He had a 23-year-old maternal half-brother who was healthy. His physical examination revealed normal growth parameters, single palmar crease, and distinctive facial features including long eyelashes, upper eyelid fullness, smooth philtrum, dental crowding, pointed chin, and tubular nose. His mother was 46 years old and reported to abuse drugs and to have psychiatric illness (Table 1).

Family 5 includes three sisters and their mother. The proband was the youngest sister who was 4 years old and presented with right lower extremity hemihyperplasia. She was born at term with birth weight of 3.4 kg. During pregnancy, the mother developed gestational diabetes that was diet-controlled. At the age of 6 months, the proband was noticed to have a longer right leg than the left. Subsequent X-rays demonstrated longer right femur and tibia when compared to the left side. She was otherwise healthy. She did not show any features of Beckwith-Wiedemann syndrome and methylation studies for Beckwith-Wiedemann syndrome were also normal. She had normal development and no behavioral problems. Physical examination showed asymmetry of lower extremities with the right leg fuller and longer than the left. Growth parameters were normal and distinctive facial features were noticed (Figure 1C). The middle sister was 6 years old who had learning difficulties, ADHD, and visual processing deficits. She was moved from 1st grade back to kindergarten and required special tutoring. She had distinctive facial features (Figure 1D). The oldest sister was 8 years old and had normal development and excellent school performance. She had distinctive facial features (Figure 1E). They did not have other siblings. The mother was 39 years old and has normal cognition with no learning difficulties. She had a college degree. She had distinctive facial features (Figure 1F) (Table 1).

Family 6 includes a girl and her mother. The proband was an 11 year old female presented with overweight. She was born at term with uncomplicated perinatal course and birth weight of 3.7 kg. Her weight had been above the 95^th^ percentile ever since the age of 9 months. She had normal development during infancy and early childhood. She did not have any learning difficulties and had an excellent school performance. Her medical history was significant for tympanostomy tube placement at the age of 2 years. Previous evaluation included methylation test for Prader-Willi syndrome, thyroid function test, leptin level, melanocortin 4 receptor gene sequencing, cortisol level, bone age, and HbA1c were all normal. Her physical examination revealed a weight that is about 4SD above the mean, height at the 90^th^ percentile, head circumference at the 95^th^ percentiles, normal hand and feet length, a single café au lait spot on her back, and some distinctive facial features (Figure 1G). She had a 17 year-old sister who is healthy and not overweight. Her mother who was 47 years old was healthy, but overweight. The mother had a college degree and reported no learning difficulties or psychiatric illnesses. The mother had one spontaneous miscarriage at 12 weeks gestational age.

Family 7 includes a 3 month-old female infant who was born at term with uncomplicated perinatal course. During pregnancy, the mother underwent chorionic villus sampling (CVS) for advanced maternal age and karyotype and array CGH were performed. The proband was healthy with normal physical examination. She had a healthy 2-year-old brother. The mother was 37 years old with no significant medical history.

### Array CGH and FISH analyses

The proband of family 1, the two brothers of family 2, the probands of family 3 and 4, and the three sisters of family 5 were all found to have ~0.5 Mb Xq28 duplications spanning from 154.1 to 154.6 Mb based on hg19 (153.7 - 154.2 Mb based on hg18) by array CGH and confirmed by FISH analysis. The mothers in families 1, 2, and 5 were also found to carry the same duplication by FISH analysis. The mothers in families 3 and 4 were unavailable for testing.

The proband of family 6 was found to have ~0.5 Mb Xq28 deletion spanning from 154.1 to 154.6 Mb (hg19) by array CGH and confirmed by FISH analysis. Her mother was found to carry the same deletion by FISH analysis. Her sister, maternal grandfather, and maternal aunt were also tested and found not to carry the deletion. Her maternal grandmother was deceased. The proband of family 7 was found to carry the same ~0.5 Mb Xq28 deletion by array CGH and confirmed by FISH analysis on the CVS sample that was obtained prenatally. Parental array CGH did not show the deletion indicating that this deletion is *de novo* in the proband.

### Chromosome X inactivation assay

The mother in family 1, the mother in family 2, and the oldest sister and the mother in family 5 showed skewed XCI, whereas the youngest and the middle sister in family 5 showed random XCI. The results of the XCI performed at the *AR* locus were suggestive of preferential inactivation of the chromosome X carrying the duplication in the mothers of families 1 and 5. However, the XCI at the *FMR1* locus results was suggestive that the normal chromosome X is preferentially inactivated in the mothers in families 1, 2, and 5 (Table 2). Both the proband and her mother in family 6 showed 95% skewed patterns. The proband of family 7 showed 100% skewing.

**Table 2:** **Chromosome X inactivation in females with** ***int22h1/int22h2*** **-mediated Xq28 duplication**

		**El-Hattab** ***et al.*** **2011 (Ref** **[** 7 **]** **)**			**Lannoy** ***et al.*** **2013** **(Ref** **[** 8 **]** **)**	**Vanmarsenille** ***et al.*** **2014 (Ref** **[** 9 **]** **)**		**Mother in family 1**	**Mother in family 2**	**Family 5**			
		**Mother in family 1**	**Mother in family 2**	**Mother in family 3**	**Mother of case 3**	**Mother in family T61**	**Mother of AV1**			**Youngest sister**	**Middle sister**	**Oldest sister**	**Mother**
**Cognitive impairment**		+	+	+	-	+	-	+	-	-	+	-	-
**XCI**		Skewed	Skewed	Skewed	Skewed	Random	Skewed	Skewed	Skewed	Random	Random	Skewed	Skewed
**AR locus**	Allele size	**239**/248	**254**/242	242		320/341	**317**/314	**266**/239	**254**/230	236/239	236/239	**236**/239	**239**/266
	Ratio	89:11	88:12	NA	80:20	68:32	89:11	99:1	95:5	50:50	62:38	80:20	88:12
	Comments	Both sons with Xq28 duplication had the 248 allele suggesting that the normal chromosome X (239) is preferentially inactivated in the mother	Son with Xq28 duplication had the 242 allele suggesting that the normal chromosome X (254) is preferentially inactivated in the mother				Son with Xq28 duplication had the 317 allele suggesting that the duplicated chromosome X (317) is preferentially inactivated in the mother	Son with Xq28 duplication had the 266 allele suggesting that the duplicated chromosome X (266) is preferentially inactivated in the mother	One son had the 230 allele and the other had the 254 allele	Allele 239 is shared among all suggesting that this allele is the duplicated chromosome and the normal chromosome X (236) is preferentially inactivated in the older sister while the duplicated chromosome X (239) is preferentially inactivated in the mother.			
**FMR1 locus**	Allele size	**380**/281	**308**/278	**314**/308				**293**/328	**322**/328	322:325	322	322	**328**:322
	Ratio	87:13	87:13	94:6				93:7	95:5	50:50	NA	NA	81:19
	Comments	One son had the 281 allele whereas the other had the 380 allele	Son with Xq28 duplication had the 278 allele suggesting that the normal chromosome X (308) is preferentially inactivated in the mother	Son with Xq28 duplication had the 314 allele suggesting that the duplicated chromosome X allele (314) is preferentially inactivated in the mother				Son with Xq28 duplication had the 328 allele suggesting that the normal chromosome X (293) is preferentially inactivated in the mother	Both sons with Xq28 duplication had the 328 allele suggesting that the normal chromosome X (322) is preferentially inactivated in the mother	Allele 322 is shared among all suggesting this allele is the duplicated chromosome and the normal chromosome X (328) is preferentially inactivated in the mother.			

The preferentially inactive alleles are in bold.

### Immunological work up

Because of the recurrent infections observed in the males with Xq28 duplication, an immunological evaluation was performed for the probands of families 1, 3, and 4 and the two brothers in family 2. Immunological work up, which included immunoglobulin levels (IgG, IgM, IgA), tetanus toxoid antibody level, *H. influenzae* type b antibody level, lymphocyte subset (T-cell, B-cells, NK cells) panel, and lymphocyte mitogen and antigen stimulation did not reveal any significant abnormalities.

## Discussion

The expanded use of high-resolution genome analysis by array CGH has led to the identification of several new microdeletion and microduplication syndromes [12,17]. Herein we present new families and review previously reported ones with the newly described *int22h1/int22h2*-mediated Xq28 duplication syndrome. Before this report, molecularly-confirmed *int22h1/int22h2*-mediated Xq28 duplications were described in only 15 individuals (Table 1) [7-9]. In this report, we present 11 additional individuals with *int22h1/int22h2*-mediated Xq28 duplication including 5 males and 6 females. The 5 cognitively impaired males presented with behavioral problems, recurrent upper respiratory tract infections, atopic diseases, and distinctive facial features. The 6 females exhibited a milder phenotype with mild cognitive impairment in the form of learning difficulties in two, ADHD in one, and some distinctive facial features similar to the affected males (Table 1).

The similarity of clinical features of the families in this report with the previously reported families supports the notion that *int22h1/int22h2*-mediated Xq28 duplication causes a recognizable syndrome that affects males with females exhibiting a milder phenotype. Cognitive impairment occurs in all males with *int22h1/int22h2*-mediated Xq28 duplication syndrome. Common features in males are behavioral problems (ADHD and aggressiveness), recurrent upper respiratory tract infections, and distinctive facial features (high forehead, long face, upper eyelid fullness, deep seated eyes, broad nasal bridge, anteverted nares, long philtrum, thick lower lip, microretrognathia, and large ears). Less common features are obesity, tall stature, and atopic diseases. Females manifest a milder phenotype with cognitive impairment in the form of learning difficulties being observed in the majority. Minor distinctive facial features similar to affected males can be observed. None of the females had recurrent infections or atopic diseases. Only one girl was reported to have ADHD. Several congenital malformations have been sporadically described such as esophageal atresia with tracheoesophageal fistula, Pierre-Robin sequence, cardiovascular malformation, and hemihyperplasia. Because of the small number of reported cases it is difficult to judge whether these malformations are related to the chromosomal aberration or just coincidences.

Recurrent upper respiratory tract infection is a common feature in males with *int22h1/int22h2*-mediated Xq28 duplication syndrome occurring in 5/9 previously reported males and in all the five males in this report. We have performed immunological evaluation for these 5 males, however, the results did not show any significant abnormalities excluding major defects in cellular immunity and antibody production.

The *int22h1/int22h2*-mediated Xq28 duplications were confirmed to be maternally inherited in all previously reported families except one family in which the mother refused blood sampling [7-9]. In families 1, 2, and 5 in this report the duplications were also confirmed to be maternally inherited, whereas the mothers in families 3 and 4 were unavailable for testing. Therefore, this duplication has been shown to be maternally inherited for all the subjects reported so far whose mothers were tested.

XCI analyses performed in the 6 previously reported females and the 6 females in this report revealed skewed XCI in 9 out of 12 females with *int22h1/int22h2*-mediated Xq28 duplication (Table 2). XCI assays provided inconsistent results regarding whether females with skewed XCI inactivate the normal chromosome X or the one carrying the duplication. Additionally, there has been no clear correlation between the XCI pattern and the cognitive phenotypes with random and skewed XCI occurring in both cognitively normal and impaired females as detailed in Table 2. Peripheral leukocytes are used for the XCI assays; therefore, the results exclusively reflect the XCI status of hematopoietic cells. It is possible that a different XCI pattern occurs in the nervous system. Such tissue-specific variation of XCI patterns may provide a feasible explanation of the observed lack of correlation between the results of XCI pattern in leukocytes and the cognitive phenotype [16,18]. A possible explanation for the inconsistent predictions of the inactivated chromosome X is the occurrence of a recombination event between the tested locus and the Xq28 duplication region which can result in the translocation of the duplication region to the opposite allele yielding results contrary to the actual situation.

The duplication breakpoints are localized to the directly-oriented LCRs *int22h1* located within intron 22 of the *F8* gene and *int22h2* situated ~0.5 kb telomerically to *int22h1*. A third homologous region *int22h-3* is located ~0.6 kb telomerically to *int22h1*. Genomic inversions between *int22h-1* and either *int22h-2* or *int22h-3*, disrupts the *F8* gene in nearly half of severe hemophilia A cases [19]. However, *int22h1/int22h2*-mediated Xq28 duplication does not result in hemophilia A because a complete copy of *F8* gene is preserved after formation of this duplication [7]. None of the individuals reported here with *int22h1/int22h2*-mediated Xq28 duplication demonstrated any bleeding tendency.

**Figure 2:**
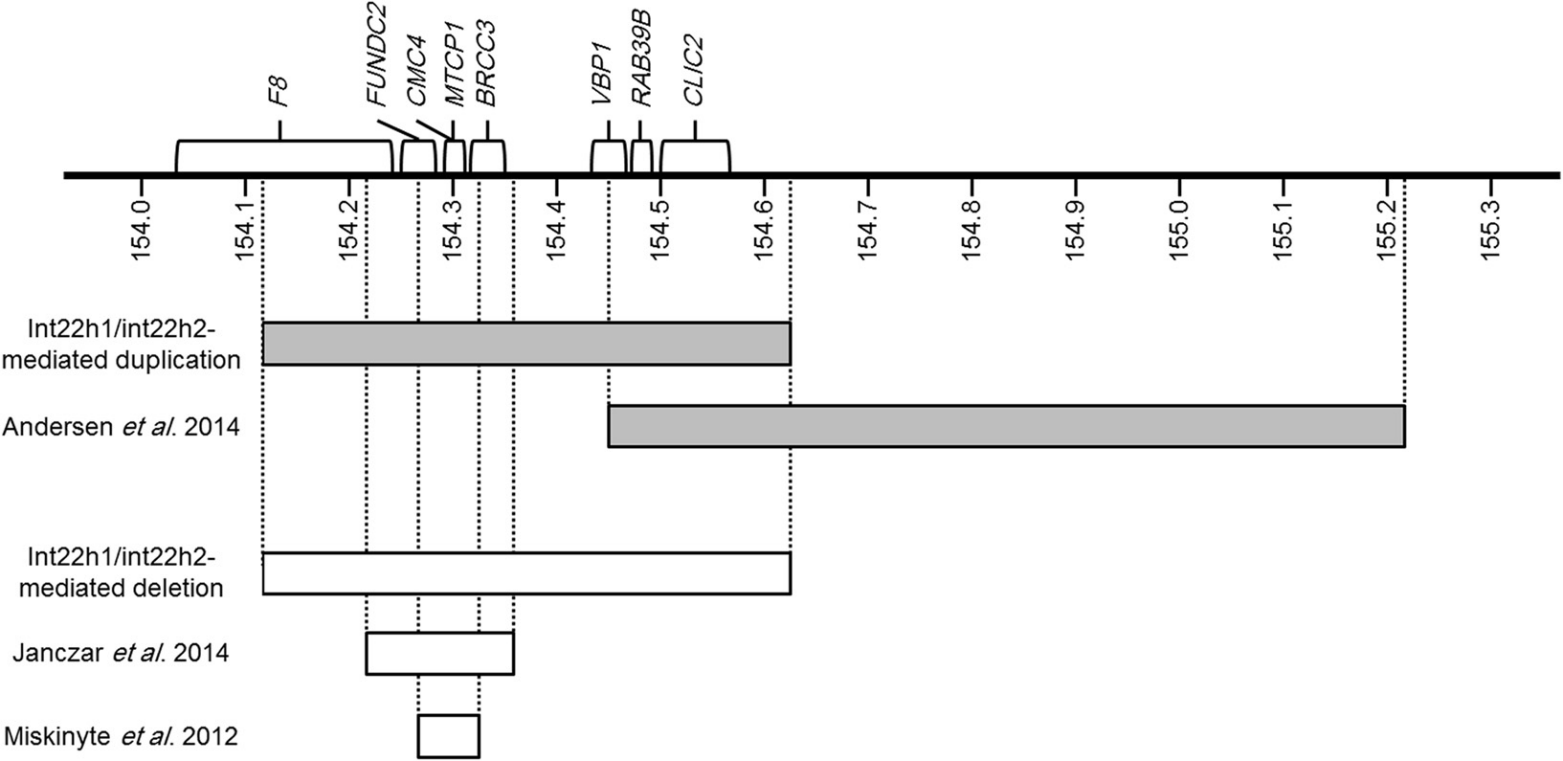
**Schematic representation of the Xq28 region (154.0 – 155.3 Mb).** Duplications are displayed as gray and deletions as white rectangles. Genes in the *int22h-1/int22h-2*-mediated Xq28 rearrangement region are displayed at the upper panel. The nucleotide position numbers are in Mb based on hg19. Andersen *et al.* 2014 is reference [26], Janczar et al. 2014 is reference [28], and Miskinyte et al. 2012 is reference [27].

In addition to part of *F8* which encodes the coagulation factor VIII, other genes in the ~0.5 Mb duplicated region between *int22h1* and *int22h2* are: *FUNDC2, CMC4, MTCP1, BRCC3, VBP1, RAB39B,* and *CLIC2* (Figure 2). The cognitive impairment in individuals with *int22h1/int22h2*-mediated Xq28 duplication is likely due to increased dosage of one or more of the genes in the duplicated region resulting in altered neuronal homeostasis. The most likely candidate gene is *RAB39B*. The *RAB39B* gene encodes a member of the Rab protein family, which are small GTPases involved in intracellular signaling proteins that coordinate vesicle trafficking during a variety of cellular processes, including neuronal development and signaling [20,21]. Loss-of-function mutations in *RAB39B* have been identified in families with XLID. It has also been demonstrated in mouse model that knocking down of *Rab39b* in primary hippocampal neurons impairs synapse formation and neuronal differentiation and maturation indicating a key role for *RAB39B* in normal neuronal functioning [22]. On the other hand, *RAB39B* overexpression was found in 2 subjects with *int22h1/int22h2*-mediated Xq28 duplication. Overexpression of *Rab39b* in mouse primary hippocampal neurons results in decreased neuronal branching and synapse number, suggesting that the increased dosage of *RAB39B* causes a disturbed neuronal development [9]. Another candidate gene that may contribute to the phenotype is *CLIC2*. The *CLIC2* gene encodes chloride intracellular channel 2 (CLIC2) protein that functions as an intrinsic stabilizer of ryanodine receptors (RyR) therefore it can modulate calcium signaling through the regulation of RyR channel activity [23,24]. A missense mutation in *CLIC2* was reported in two brothers with cognitive impairment, seizures and cardiac anomalies [25]. The suggestion of *RAB39B* and *CLIC2* being responsible for the observed phenotype in *int22h1/int22h2*-mediated Xq28 duplication syndrome is further supported by the identification of an overlapping ~0.8 Mb duplication in Xq28 in individuals having clinical features similar to those observed in *int22h1/int22h2*-mediated Xq28 duplication syndrome [26]. This ~0.8 Mb Xq28 duplication (154.4 – 155.2 Mb, hg19) overlaps with the *int22h1/int22h2*-mediated Xq28 duplication in a region that only harbors *RAB39B* and *CLIC2* (Figure 2), and has recently been reported in three siblings with cognitive impairment, behavioral problems (ADHD and aggressiveness), short stature, and distinctive facial features including high forehead, hypertelorism, broad nasal bridge, thin upper lip, and cupped ears. The three siblings had an additional deletion that removes a segment of the pseudoautosomal region of Xp22.33 including *SHOX* explaining the observed short stature in these siblings [26]. The considerable phenotypic overlap between these siblings with this ~0.8 Mb Xq28 duplication and individuals with *int22h1/int22h2*-mediated Xq28 duplication suggests that the genes within the shared region, *RAB39B* and *CLIC2*, contribute to the phenotype in both groups [26].

The mother and daughter in family 6 with the reciprocal *int22h-1/int22h-2*-mediated Xq28 deletion had extremely skewed XCI patterns and normal cognition, supporting the idea that this deletion has no phenotypic effect in females [7]. The proband in family 7 is the first reported case with prenatally diagnosed and *de novo int22h1/int22h2*-mediated deletion. This infant adds more evidence to the benign nature of this deletion in females. Females carrying the *int22h1/int22h2*-mediated Xq28 deletions should not exhibit clinical signs of hemophilia A as a result of the preferential inactivation of the chromosome X harboring the *F8* gene-inclusive deletion [7]. None of the individuals in families 6 and 7 show any signs of bleeding tendency supporting that.

It has been also suggested that the *int22h-1/int22h-2*-mediated Xq28 deletion is embryonic lethal in males resulting in higher miscarriage rates in females carrying this deletion [7]. The previously reported mother with this deletion had two spontaneous miscarriages [7] and the mother of family 6 had one spontaneous miscarriage, which may be related to the deletion. The proposed lethality in male embryos carrying the deletion suggests that one or more of the genes in the deleted region (*F8, FUNDC2, CMC4, MTCP1, BRCC3, VBP1, RAB39B,* and *CLIC2*) may be very essential for early development and the hemizygous loss of this or these genes is incompatible with life. Deletions including *FUNDC2, CMC4, MTCP1,* and *BRCC3* were reported in individuals with syndromic Moyamoya disease, characterized by angiopathy, short stature, and distinctive facial features (Figure 2) [27]. Recently, an ~150 kb deletion of Xq28 encompassing part of *F8, FUNDC2, CMC4, MTCP1,* and *BRCC3* was reported in a child with severe hemophilia A, Moyamoya disease, and distinctive facial features (Figure 2) [28]. These two reports provide evidence that the loss of these 5 genes is compatible with life. From the remaining three genes, loss-of-function mutations in both *RAB39B* and *CLIC2* have been reported in individuals with cognitive impairment making it less likely that the hemizygous loss of these genes results in the lethality in males with *int22h1/int22h2*-mediated deletion [22,25]. The remaining gene is *VBP1* which encodes the Von Hippel-Lindau binding protein-1 (VBP1). Studies of double-stranded RNA interference in *C. elegans* against VBP1 messenger resulted in an arrest of embryogenesis at morula stages, suggesting that this protein is necessary for morphogenesis [29]. Therefore, the hemizygous loss of *VBP1* may be the most likely cause of male embryonic lethality in *int22h1/int22h2*-mediated deletion.

## Conclusions

In conclusion, *int22h1/int22h2*-mediated Xq28 duplication causes a recognizable syndrome in males, with females exhibiting milder phenotypes. Cognitive impairment occurs in all males. Common features in males are behavioral problems, recurrent upper respiratory tract infections, and distinctive facial features. This duplication has been shown to be maternally inherited for all the subjects whose mothers were tested. Skewed XCI has been observed in the majority of females carrying the duplication. Increased dosage of one or more of the genes in the duplicated area may alter neuronal homeostasis, resulting in the observed cognitive impairment. *RAB39B* and possibly *CLIC2* are the most likely candidate genes in which mutations have been reported in individuals with cognitive impairment. The reciprocal *int22h1/int22h2*-mediated Xq28 deletion results in extremely skewed XCI patterns and no clinical phenotype in females. It is suggested that this deletion is embryonic lethal in males; therefore females carrying this deletion may be at higher risk of miscarriages.
